# Ventricular fibrillation induced by atrial threshold search: a case report

**DOI:** 10.1093/ehjcr/ytaf131

**Published:** 2025-03-15

**Authors:** David Sprenkeler, Ferry Hersbach, Ad Oomen, Clara van Ofwegen-Hanekamp, Mathias Meine

**Affiliations:** Department of Cardiology, Division of Heart & Lungs, University Medical Center Utrecht, Heidelberglaan 100, 3584 CX Utrecht, the Netherlands; Department of Cardiology, Diakonessenhuis, Bosboomstraat 1, 3582 KE Utrecht, the Netherlands; Department of Cardiology, Diakonessenhuis, Bosboomstraat 1, 3582 KE Utrecht, the Netherlands; Department of Cardiology, Diakonessenhuis, Bosboomstraat 1, 3582 KE Utrecht, the Netherlands; Department of Cardiology, Division of Heart & Lungs, University Medical Center Utrecht, Heidelberglaan 100, 3584 CX Utrecht, the Netherlands

**Keywords:** Implantable cardioverter defibrillator, Ventricular fibrillation, Pro-arrhythmia, Case report

## Abstract

**Background:**

Implantable cardioverter defibrillators (ICDs) have been proven to reduce the risk of sudden cardiac death from ventricular tachyarrhythmias. However, ICDs can sometimes induce malignant arrhythmias. We describe a case of ventricular fibrillation triggered by an automatic atrial threshold search.

**Case summary:**

A 72-year-old man presented after a syncopal episode. His medical history included moderate aortic regurgitation and a symptomatic second-degree atrioventricular (AV) block, for which he received a dual-chamber pacemaker in 2013, later upgraded to a CRT-D due to pacing-induced heart failure. ICD interrogation revealed an episode of ventricular fibrillation terminated by a shock. The arrhythmia started directly after an atrial threshold search. Extensive work-up did not reveal a cause of the arrhythmia, therefore, we considered it most likely that the atrial threshold test triggered the ventricular fibrillation. Atrial Capture Management was disabled, and the patient was discharged. No further ventricular arrhythmias or ICD therapies were observed.

**Discussion:**

Automatic threshold measurement algorithms are intended to ensure effective myocardial capture and enhance safety but can sometimes inadvertently cause arrhythmias. The underlying mechanism in this case may be related to the switch from biventricular to right ventricular (RV)-only pacing during Atrial Capture Management, which increases dispersion in repolarization, facilitating early afterdepolarizations and triggering polymorphic tachycardias. Notably, newer ICD models mitigate this risk by maintaining biventricular pacing during this test. This case underscores the need for careful programming and monitoring of ICD algorithms.

**Learning points**

- Pro-arrhythmia induced by cardiac implantable electronic devices is a rare but serious complication.
- Even seemingly innocent algorithms such as an atrial threshold search can result in life-threatening arrhythmias
- One should therefore programme these algorithms carefully, especially reconsidering the need in patients with low and stable threshold levels.

## Introduction

The implantable cardioverter defibrillator (ICD) has been demonstrated in multiple randomized clinical trials to reduce the risk of sudden cardiac death caused by ventricular tachyarrhythmias.^1–3^ Consequently, European guidelines recommend prophylactic implantation of an ICD in patients at increased risk of sudden cardiac death.^4^ However, in rare instances, the anti-tachycardia therapy provided by these devices may paradoxically induce malignant arrhythmias. This device-induced pro-arrhythmia is most often due to inappropriate device programming or device malfunction, such as inappropriate therapy to supraventricular arrhythmias or oversensing of non-physiologic signals.^5^ Modern cardiac electronic implantable devices are equipped with advanced algorithms that automatically assess key functionalities, such as lead integrity, sensing, and pacing thresholds. While these algorithms are intended to enhance the safety and efficacy of device therapy, they can occasionally have deleterious adverse effects. In this report, we describe a case of ventricular fibrillation triggered by an automatic atrial threshold search.

## Summary figure

**Figure:**
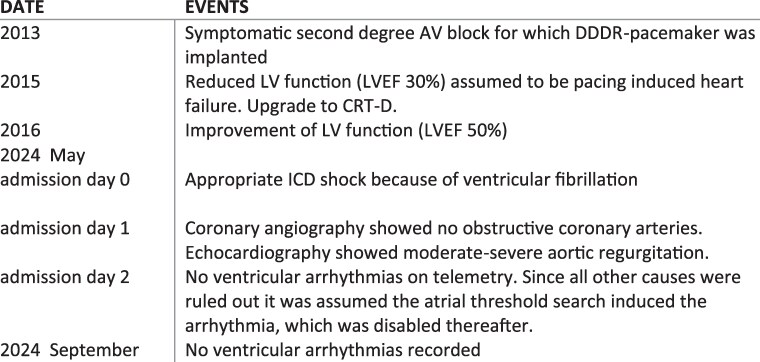


## Case presentation

**Figure 1:**
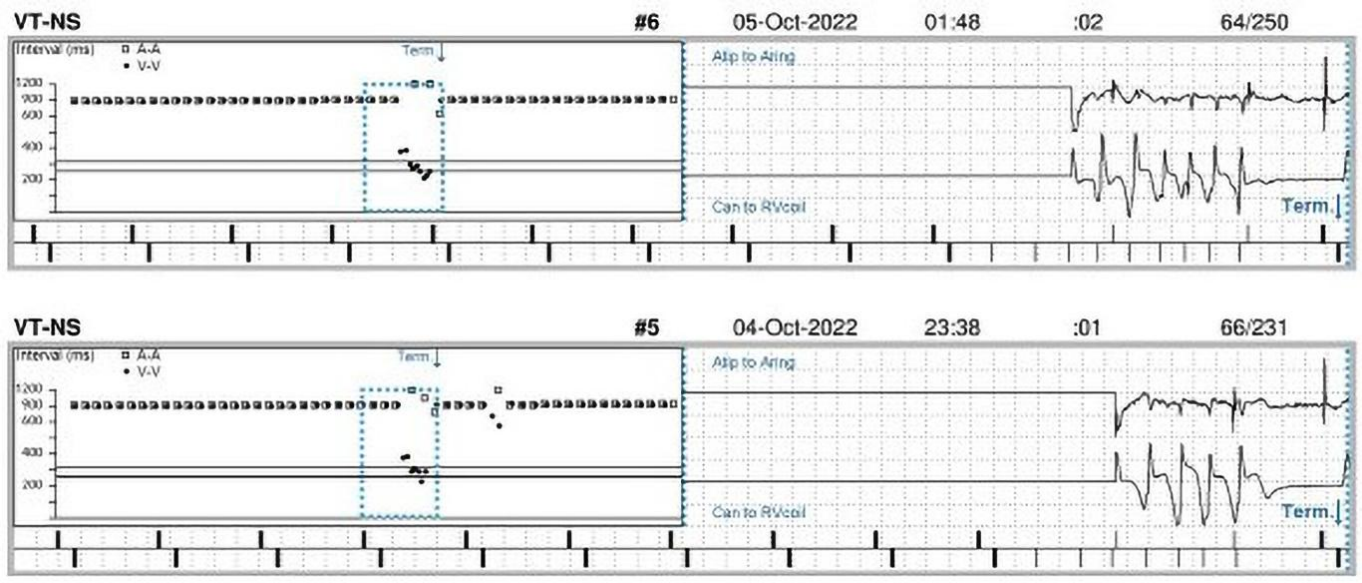
Intracardiac electrogram of non-sustained ventricular tachycardia.

A 72-year-old man presented to the emergency department following a syncopal episode in the bathroom. His medical history included a moderate aortic regurgitation and a symptomatic second-degree atrioventricular (AV) block for which he had received a dual-chamber pacemaker (Biotronik Evia-DRT™) in 2013. He had no other comorbidities and did not smoke. Two years after pacemaker implantation, his device was upgraded to a CRT-D (Medtronic Amplia MRI^™^) due to pacing-induced heart failure. Coronary angiography excluded obstructive coronary artery disease as the cause of reduced left ventricular function. During follow-up, his left ventricular ejection fraction (LVEF) improved to approximately 50%, and he had no anti-tachycardia pacing or ICD shocks, although short runs of non-sustained ventricular tachycardia were noted during device interrogation (*Figure 1*). His medications included ivabradine 5 mg b.i.d. and losartan 50 mg o.d.

**Figure 2:**
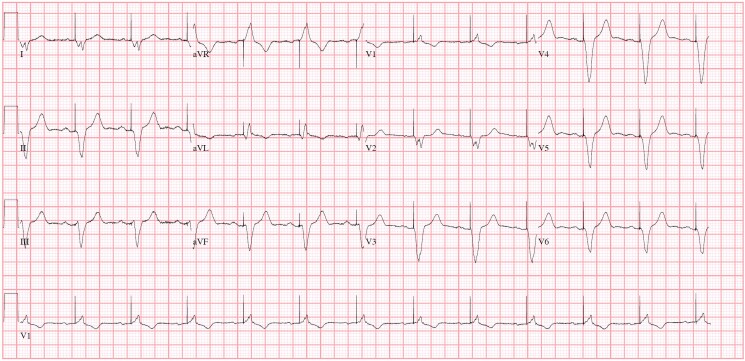
12-Lead ECG showing an atrial sensed, biventricular paced rhythm of 74 b.p.m. with no repolarization abnormalities.

**Figure 3:**
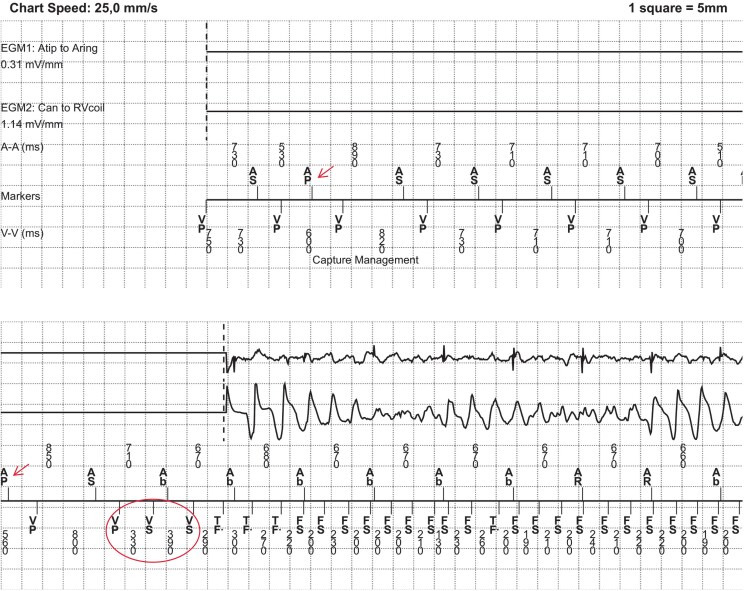
Intracardiac electrograms of VT/VF episode. Atrial electrogram (upper tracing), far-field RV electrogram (middle tracing) and marker channel (lower tracing) are shown. Prior to the arrhythmia, atrial threshold search is performed (as denoted by ‘Capture Management’). Atrial sensing (AS) with RV pacing (VP) with a long AV interval of 240 ms (programm-sensed AV delay 120 ms) is interrupted by atrial pacing (AP) with a shorter coupling interval (around 500 ms) to test for atrial capture (arrows). This sequence is followed by two short-coupled premature complexes (330 and 390 ms) which initiate ventricular fibrillation (circle). After detection by the device, the arrhythmias is successfully terminated by a 35J shock (not shown).

**Figure 4:**
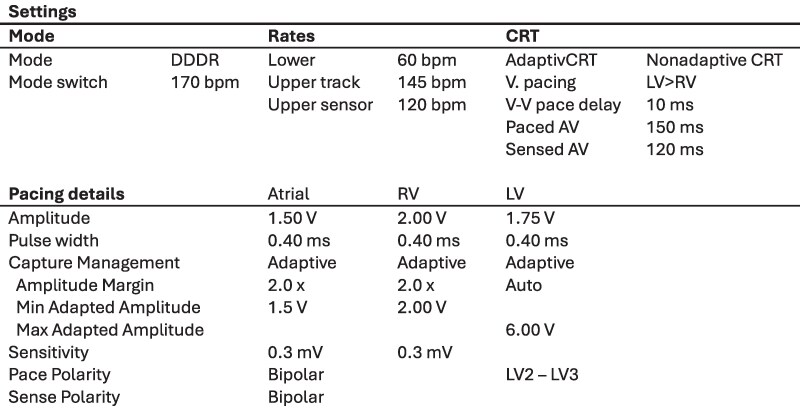
Device settings. Note that Capture Management is enabled for atrial, RV and LV channel.

Before and after the syncope, he reported no cardiac symptoms such as chest pain or palpitations. He had no family history of sudden cardiac death. Physical examination at the emergency department was unremarkable. His electrocardiogram showed a biventricular paced rhythm of 65 b.p.m. with a QTc of 470 ms and no new repolarization abnormalities. (*Figure 2*) ICD interrogation revealed an episode of ventricular fibrillation terminated by a 36J defibrillation shock (*Figure 3*). Device settings are shown in *Figure 4*.

When reviewing the tracing, an atrial threshold search, known as Atrial Capture Management™, was noted on the marker channel prior to the onset of the arrhythmia. One method used for evaluating atrial capture is Atrial Chamber Reset (ACR). During stable sinus rhythm, an atrial test pace is given with a shorter cycle length. If there is no capture, the sinus node is not reset, and the P wave will occur in the AV interval, resulting in an atrial refractory-sensed event (denoted by AR). If the atrial pace captures the myocardium, no AR is seen in the AV interval, and the device concludes effective capture. During the test, this type of CRT-D switches from biventricular pacing to right ventricular (RV)-only pacing, denoted by VP instead of BV in the marker channel.

To safe battery life, pre-arrhythmia EGM is standard disabled in Medtronic ICD’s, complicating the determination of the arrhythmia’s exact onset mechanism. However, in this case the arrhythmia starts directly after the atrial threshold search, suggesting a causal relationship.

Further work-up of the arrhythmia's aetiology was conducted. Laboratory results showed a mildly increased troponin of 15.5 ng/L (normal values < 14 ng/L), which did not rise further during hospital admission. Electrolytes were within normal limits. A new echocardiogram showed mildly reduced LV function (LVEF 45%) with moderate to severe aortic regurgitation. Coronary angiography showed no obstructive coronary artery disease. With all other causes ruled out, it was hypothesized that the atrial threshold test might have triggered the ventricular fibrillation. Consequently, Atrial Capture Management™ was turned off, and the patient was discharged the following day with a repeat echocardiogram scheduled in 6 months to follow-up on the aortic regurgitation. No sustained ventricular arrhythmias or ICD therapy have been observed since.

## Discussion

Automatic threshold measurement algorithms are designed to enhance battery longevity and improve patient safety by ensuring effective myocardial capture despite fluctuating capture thresholds. To our knowledge, only one other case has been reported where atrial threshold search with a Medtronic ICD resulted in pro-arrhythmia.^6^ In that case, the switch from biventricular to RV-only pacing during Atrial Capture Management™ led to late premature ventricular complexes (PVCs) that coincided with ventricular pacing and fell in the ventricular blanking period. These PVCs were not displayed on the marker channel and were only identified through telemetry monitoring. This led to multiple episodes of polymorphic ventricular tachycardia and ventricular fibrillation, requiring several ICD shocks.

The similarities with our case and the report of Kristensen *et al*. are remarkable. Both started during stable sinus rhythm of around 80 b.p.m. with no (sensed) ventricular ectopy before the start of the arrhythmia. In our case we do not have recordings such as telemetry other than the marker channel from the device. Therefore, we do not know if late ectopic beats were not sensed, although they would have to occur exactly at the same time as ventricular pacing to fall in the blanking period (with the current settings within 200 ms after VP). The PVC that initiates the arrhythmia is short coupled (330 ms) with a relatively longer preceding interval (800 ms), suggesting a short-long-short-like mechanism. The electrophysiological mechanism of the pro-arrhythmic consequence of the atrial threshold search is unknown but may relate to the acute change in ventricular activation sequence when switching from biventricular to RV pacing and its effects on repolarization and refractoriness. Altering the depolarization sequence, either from intrinsic conduction to pacing or from BiV pacing to RV-only pacing as in this case, increases dispersion in repolarization which facilitates propagation of early afterdepolarizations that can induce polymorphic ventricular tachycardias or torsade des pointes, especially in patients at risks of these arrhythmias, such as in heart failure, long QT syndrome or when using QT-prolonging drugs.^7^ Several case reports of ventricular tachycardia and electrical storms immediately following CRT have been published.^8–10^ Furthermore, RV pacing has also been associated with non-sustained VT and ICD shocks.^11,12^ Interestingly, there appears to be a bimodal relationship between the percentage of RV pacing and arrhythmia burden; patients with <1% or >98% RV pacing have the lowest risk for ventricular arrhythmias, implying that intermittent changes in activation sequence are especially pro-arrhythmic. Interestingly, physiological pacing, such as His bundle pacing or left bundle area pacing, appears to have preserved dispersion of repolarization, as measured by *T*_peak_ – *T*_end_, which may reflect a lower arrhythmic risk.^13,14^Thus, a sudden change from biventricular to RV pacing, as in this case, may have a pro-arrhythmic effect and can, under certain circumstances, induce life-threatening arrhythmias. Notably, newer generation Medtronic devices use biventricular pacing during Atrial Capture Management™, thereby reducing the risk of these arrhythmias. Physicians must therefore consider proper ICD-programming other than nominal settings, especially the need for specific algorithms such as Atrial Capture Management™, which could have deleterious consequences in certain patients.

## Conclusion

Algorithms such as Atrial Capture Management™ are designed to improve device functionality and patient safety but can in rare instances have the opposite effect and be pro-arrhythmic. This case highlights the importance of careful ICD-programming, especially reconsidering the need for these specific algorithms in patients with low and stable thresholds.

## Data Availability

All the identified data are available upon request.

## Lead author biography

David Sprenkeler was born on the 2nd of May, 1990 in Amsterdam, the Netherlands. He received his Bachelor degree in Medicine in 2011 at Utrecht University. During the Master program of Medicine, he obtained a specific interest in the field of Cardiology. After obtaining his medical degree in 2014, he started a PhD trajectory at the Department of Medical Physiology and Cardiology. He defended his thesis in 2018. Since January 2019 he is a resident in training in Cardiology in the UMC Utrecht.

## Notes

**Consent:** Written informed consent for publication of this case was obtained in compliance with COPE guidelines.

**Funding:** No funding was needed for this manuscript.
